# Effects and clinical feasibility of a behavioral treatment for sleep problems in adult attention deficit hyperactivity disorder (ADHD): a pragmatic within-group pilot evaluation

**DOI:** 10.1186/s12888-019-2216-2

**Published:** 2019-07-24

**Authors:** Susanna Jernelöv, Ylva Larsson, Milagros Llenas, Berkeh Nasri, Viktor Kaldo

**Affiliations:** 1 Division of Psychology, Department of Clinical Neuroscience, Karolinska Institutet, Stockholm, Sweden; 2 Centre for Psychiatry Research, Department of Clinical Neuroscience, Karolinska Institutet, & Stockholm Health Care Services, Stockholm County Council, Stockholm, Sweden; 3 Department of Psychology, Faculty of Health and Life Sciences, Linnaeus University, Växjö, Sweden

**Keywords:** ADHD, Sleep problems, Behavioral treatment, Pilot evaluation

## Abstract

**Background:**

Sleep disturbances, including insomnia, are common in adult Attention Deficit Hyperactivity Disorder (ADHD). Treatment of choice for insomnia is cognitive behavioral therapy (CBT-i), but evidence is lacking for CBT-i in patients with ADHD.

The purpose of this study was to investigate if patients with insomnia and other sleep problems, at a specialist clinic for ADHD, benefit from a group delivered behavioral treatment based on CBT-i; whether insomnia severity improves following this treatment.

**Methods:**

This pragmatic within-group pilot study with a pre to post and three-month follow-up design was set at a specialist psychiatric out-patient clinic for adult ADHD.

As an adjunct to care-as-usual at the clinic, a CBT-i-based group treatment targeting several sleep problems prevalent in the ADHD–population, was offered as 10 weekly 90-min group sessions and scheduled telephone support.

All outcome measures were subjectively reported by participants. Data analyzed with dependent t-tests according to intent-to-treat.

**Results:**

Nineteen patients (37 [SD 13.7] years; 68% female) with ADHD and subjectively reported sleep problems provided informed consent and pre-treatment measures. Patients had suffered from sleep problems for 15.3 [SD 13.4] years, 42% used sleep medications, 79% used stimulant medication(s).

At post-treatment, insomnia severity (Insomnia Severity Index; score range 0–28) had improved with 4.5 points (95% CI, 2.06–6.99, *p* = .002), at 3 months with 6.8 points (95% CI, 4.71–8.91, *p* < .0001) from pre-treatment.

**Conclusions:**

CBT-i adjusted for ADHD is promising for improving insomnia severity in adult patients at specialist psychiatric out-patient clinics, who suffer from ADHD and sleep disturbances.

**Trial registration:**

Study registered with the Regional ethical review board in Stockholm, January 13th 2016, Study id: 2015/2078–31/1. Study registered retrospectively with Clinicaltrials.org, February 21st 2019, ID: NCT03852966.

## Background

Attention Deficit Hyperactivity Disorder (ADHD) is a psychiatric disorder characterized by decreased attention, increased impulsivity and hyperactivity, and is often accompanied by deficits in vigilance and executive functioning [1]. In adults, a prevalence of ADHD of 2.5% has been reported [2]. ADHD in adults, as in children, is commonly treated with stimulant medication, but psychosocial interventions are also recommended as symptoms and impaired everyday functioning commonly persist even after pharmacological treatment [3]. As a consequence, psychological treatments to ameliorate ADHD problem areas are being developed and tested [4, 5].

Patients with ADHD often have several comorbid conditions [6], including sleep-wake problems. Adults with ADHD report more sleep problems than healthy controls [7, 8]. One study found that 82.6% of patients with ADHD had ever had sleep problems, compared to 36.5% of controls [8], and not feeling refreshed in the morning is strongly associated with ADHD [9]. Insomnia, i.e. difficulties initiating or maintaining sleep and experiencing sleep-related daytime symptoms, is highly prevalent in this patient group [10, 11]. Delayed sleep phase syndrome, i.e. a delayed circadian rhythm, is also common [12–14], and can cause difficulties with both falling asleep and waking up early. These difficulties may be due to an altered endogenous rhythm, but may also be maintained by late habits due to executive difficulties.

Despite sleep problems such as insomnia and delayed sleep phase being prevalent in adults with ADHD, very few empirical studies specifically address their treatment in this patient group. For children with ADHD, several treatments have been tried in scientific evaluations. For instance, the use of melatonin to treat sleep onset insomnia and delayed sleep phase is fairly well established [15, 16]. Unfortunately, studies on the efficacy of melatonin to treat insomnia in adult populations with ADHD are mostly lacking, but a few studies have investigated the use of light therapy with promising results [17, 18]. Even though pharmacological treatment for insomnia is commonly used, hypnotics often do not result in satisfactory levels of symptom reduction or improvement in functioning in insomnia patients [19], and the few available studies on adult ADHD are no exception [20]. Given the less than optimal effects of pharmacological treatments and the risk of adverse interactions with prescribed medications, non-pharmacological interventions may be an attractive option for the management of insomnia in patients with ADHD.

Many factors may be involved in the development of insomnia, but maintaining factors are most often behavioral [21]. Perhaps then it is not surprising that insomnia can be successfully treated with specific behavioral interventions, as has been demonstrated previously [22, 23]. In fact, cognitive behavioral therapy for insomnia (CBT-i) is now generally recommended as first line treatment, (e.g. [24–27]), and has also been shown to be effective when insomnia is comorbid with other problems, including several psychiatric problems [28, 29].

Interestingly, studies have shown that psychiatric symptoms may also improve with successful treatment of insomnia (e.g. [23, 30]). However, deteriorations have also been seen (e.g. [31]), and it is therefore important to investigate whether changes in core symptoms of ADHD may be seen after treatment of disturbed sleep.

Since sleep problems in ADHD are varied and complex, a sleep-focused CBT protocol should ideally target not only insomnia disorder according to DSM 5, but also other ADHD related sleep difficulties, such as delayed sleep phase and insufficient sleep hygiene. To our knowledge, however, no version of CBT-i has been tested in adults with ADHD as of yet.

### Aims of the study

The aim of this paper is to perform a preliminary evaluation, to investigate if insomnia severity and ADHD symptoms improve following a novel group intervention based on CBT for insomnia and adjusted for patients with ADHD (CBT-i/ADHD), and to gauge if the treatment is feasible in a clinical setting.

## Methods

### Design

This is a pragmatic within-group (i.e. non-controlled) pilot study with a pre to post design and three-month follow-up. The study was conducted in collaboration with Northern Stockholm Psychiatry, Stockholm County Council, Sweden, and set at a specialist psychiatric out-patient clinic for adults with ADHD.

The authors assert that all procedures contributing to this work comply with the ethical standards of the relevant national and institutional committees on human experimentation and with the Helsinki Declaration of 1975, as revised in 2008. The study protocol was registered and approved by the regional ethical review board in Stockholm, Sweden (Study id: 2015/2078–31/1), and written informed consent was obtained from all participants.

### Participants

The study included 19 patients at a specialist psychiatric out-patient clinic for adult ADHD. Apart from having an ADHD diagnosis and being a registered patient at the clinic, inclusion in this study required self-reported sleep problems, being interested and able to participate in the group treatment, and to return pre-treatment study questionnaires including a consent form before deadline. Medication use or comorbidities were thus not cause for exclusion, as long as the patient was able to participate in the group treatment. Patients were recruited during the fall of 2015 and spring of 2016 through referral from clinic staff, and the experimental behavioral treatment was an adjunct to care-as-usual at the clinic.

**Fig. 1:**
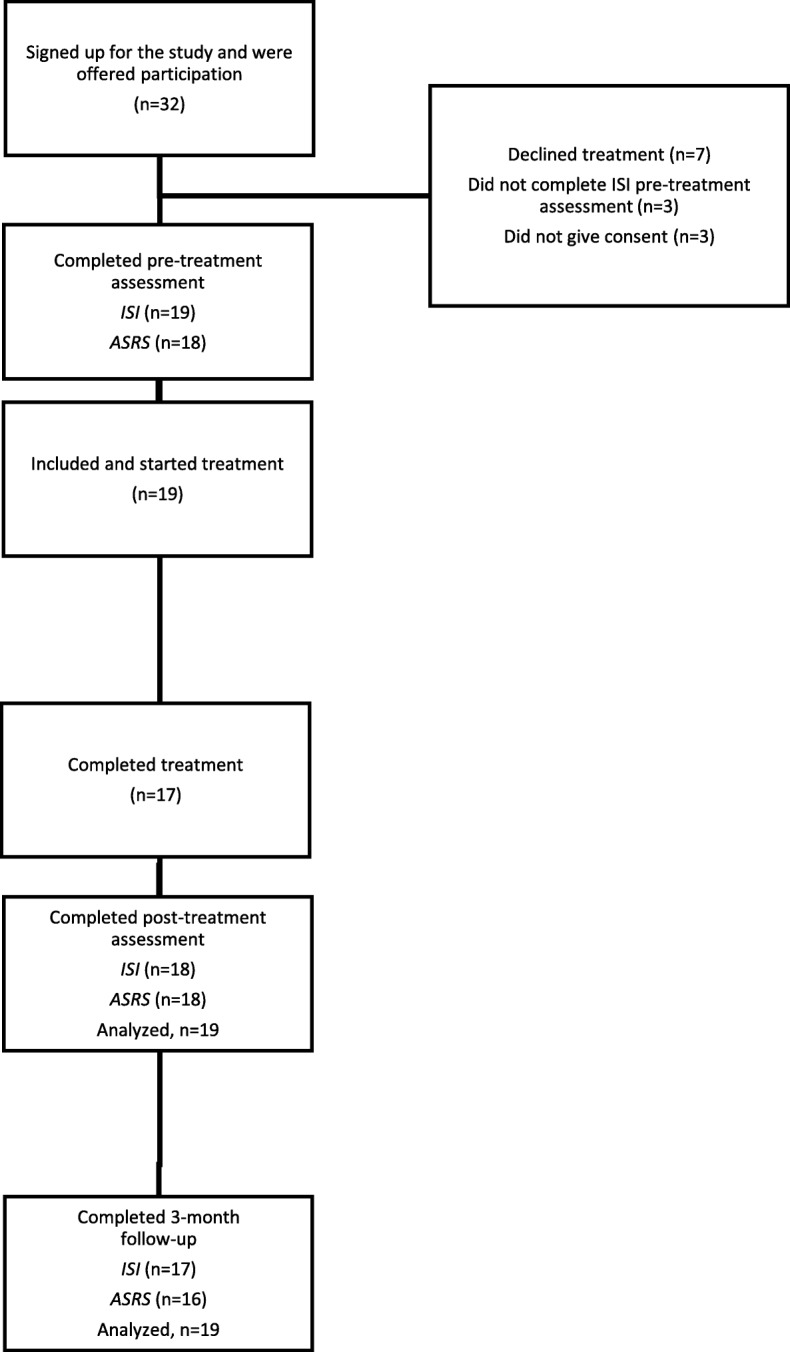
Study flow-chart

For participant flow through study, see Fig. 1.

### Procedure and assessments

Interested participants received a telephone call from study staff, with information about the study and the treatment. Patients who were still interested in participating were sent pre-treatment questionnaires to be returned before treatment start. Demographic data and other patient characteristics including diagnoses and medication use were collected at pre-treatment, also using self-report questionnaires. Outcome measures were self-rated and administered at pre-treatment, post-treatment and three-month follow-up. Treatment evaluation forms were handed out after the last treatment session and patients were asked to fill them out and return them before leaving.

#### Screening and background

The SLEEP-50 [32] was used to screen for sleep disorders. Additional background information was collected using a form with questions on age, sex, marital status, education level, and current occupation and questions on psychiatric diagnoses and problems, medication use, and morning-eveningness. More specifically, for type of ADHD, patients were asked to choose which described their type best: Predominantly inattentive, Predominantly hyperactive/impulsive, Combined subtype, or NOS; for psychiatric diagnoses an open-ended question was used; for psychiatric problems during the last year, patients were given a list and asked to mark which applied to them; medication use was gauged with an open ended questions with examples, and medications were classified according to stated use and/or substance; circadian type was measured with the question on morning-eveningness from the Karolinska Sleep Questionnaire, on a five point scale (Extreme morning type, morning type, intermediate type, evening type, extreme evening type) [33].

#### Primary symptom outcome

To evaluate insomnia severity, the Insomnia Severity Index (ISI) was used. This 7-item patient-reported questionnaire has adequate psychometric properties and is sensitive to change [34]. A 5-point scale (0–4) is used to rate each item, yielding a total score of 0 to 28. In this study, the Swedish adolescent version of the ISI (ISI-a [35]) was used. The scale has shown adequate internal reliability (Cronbach’s alpha = .83) and correspondence with other measures of insomnia [35]. Levels of insomnia severity were defined according to Bastien et al. [34]: No clinically significant insomnia (i.e. in remission): 0–7 points; Subthreshold insomnia: 8–14 points; Clinical Insomnia, moderate severity: 15–21 points; Clinical insomnia, severe: 22–28 points.

#### Secondary symptom outcome

Severity of ADHD-symptoms was measured with the Adult ADHD Self-Report Scale (ASRS), consisting of 18 items related to ADHD-symptoms (according to the DSM-IV), to be rated on a 0 (never) to 4 (very often) scale, yielding a score ranging from 0 to 72 points. The scale has two subscales for inattention and hyperactivity symptoms respectively, each 0 to 36 points. Cut off for probable ADHD is 17 points and for highly probable ADHD 24 points, on either of the subscales. The scale has shown good internal reliability (Cronbach’s alpha = .88) and good construct validity (i.e. high correlation with clinicians’ assessments of ADHD-symptoms) [36].

#### Patient compliance and satisfaction with treatment

Patient compliance was measured with (a) proportion of patients dropping out of treatment and (b) average number of group sessions attended.

Patient satisfaction with treatment was measured with a treatment evaluation questionnaire constructed for the study asking (c) if the patient would recommend this type of treatment to a friend with ADHD and sleep difficulties, and (d) the patient’s overall impression of the treatment (1–6, where 1 represents “very poor”, 2 “poor”, 3 “quite poor”, 4 “quite good”, 5 “good”, 6 “very good”). Patients were also asked about their opinion on (e) most important treatment components.

### Treatment

The treatment consisted of weekly 90-min sessions over the course of 10 weeks, and was based on CBT-i, with adjustments to both content and format. Content was adjusted by adding behavioral components targeting other sleep and circadian problems common in this patient group, such as delayed sleep phase and insufficient sleep hygiene, and by adding components targeting commonly occurring difficulties with planning and organizing, to help patients succeed in applying the sleep related treatment components (e.g. by using an alarm and a calendar, as suggested by Safren et al. [37]). Format was adjusted for instance by shortening sessions, lengthening treatment period and including a short (5–20 min) scheduled telephone call from one of the group leaders between sessions. All adjustments were made to better suit the needs of the patient group with ADHD. Regular CBT-i-components were sleep scheduling with the option of sleep compression based on sleep diary data, stimulus control, relaxation/visualization and cognitive restructuring. To work behaviorally with sleep phase problems, circadian nadir was approximated using a series of questions regarding patients’ circadian habits, for instance preferred and spontaneous rise-time, and most difficult time staying awake during the night, in order to find each patients optimal time-point for natural light exposure, meal-times and activity scheduling. For an overview of session content, please see Table 1.

**Table 1:** Session content

Session	Contents	Homework (use of strategies)
1	Introduction to treatment, information about CBT and CBT-i models of treatment. Introduction to the use of organizing-strategies. Discussing treatment goals.	Fill out a sleep diary every day. Use calendar, to-do list, distractibility reduction skills, set treatment goals.
2	Problem-solving and behavioral experiments if needed^a^. Psychoeducation about sleep, sleep myths, sleep and ADHD, and effects of ADHD medication on sleep. Role of relaxation and use of relaxation techniques.	If needed, discuss ADHD-medication timing with prescribing psychiatrist. Stabilizing sleep medication. Practicing relaxation techniques to be used both during daytime and in bed.
3	Setting the circadian rhythm. Approximating the circadian nadir of each patient and setting the appropriate “light schedule”. ^b^	Use light and darkness systematically, according to the light schedule developed in session.
4	Regularizing sleep schedule and adjusting other activities accordingly, to help set circadian rhythm and use sleep pressure to improve sleep, use of a morning-routine to get up in time. Develop each patient’s individual sleep schedule.^b^	Follow the sleep schedule developed in session (after the first week, sleep compression is applied if sleep efficiency is low), use a morning routine to get up in the morning.
5	In the evening: activity level, routines and management of pre-sleep worry. Identifying individual needs and planning how to work with them.	Gradually-less arousing activities 1,5–2 h before bedtime, “worry time” if needed, use a simple evening routine.
6	Follow up on treatment progress and goals. Stimulus control and sleep hygiene. Identifying individual sleep hygiene needs and planning how to work with it.	Getting out of bed when unable to sleep for 20 min. Follow individual plan for sleep hygiene practices.
7	Daytime activity, variability and pacing. Identifying individual needs and planning the work. Non sleep-disturbing ways to handle fatigue.	Physical activity. Increase variability of activity level, use non sleep-disturbing ways to handle fatigue.
8	Cognitive activity and sleep. Cognitive restructuring.	Identify and manage sleep disturbing thoughts.
9	Acceptance and mindfulness. Summarize the treatment, choose a strategy to apply during the last week.	Work with the chosen strategy, and try acceptance and mindfulness strategies.
10	Evaluation of treatment goals. Relapse prevention. Create an individual Sleep Plan based on strategies from the treatment.	Follow the Sleep Plan, including relapse prevention.

^a^ Problem-solving and behavioral experiments applied each following session, if needed

^b^ Continued work with both light and sleep schedule carried on to all subsequent sessions

Groups were closed, i.e. group members were the same from start to finish. Each group had between 6 and 10 participants and was led by two therapists. All therapists had undergone extensive CBT-training and training in the current treatment manual, and received weekly supervision by an experienced clinician and behavioral sleep medicine expert (SJ).

### Power considerations

The expected within-group effect size for the ISI was Cohen’s *d* = 1.0, based on previous studies of insomnia treatment in comorbid patient groups, (e g [23, 38, 39]). To achieve a power of 80%, with an alpha level of .05, 16 participants were needed. Since attrition was expected we aimed for 20 included participants.

### Data analysis

For outcome measures (ISI and ASRS), imputation for missing items in questionnaires was performed using the Corrected Item Mean substitution [40]. Data was screened for normality, and as none of the variables were statistically non-normally distributed at any of the time-points (Kolmogorov-Smirnov p’s .108 < .200), data from missing questionnaires at the post-treatment and follow-up assessments were imputed using the multiple imputation procedure in SPSS 24. Missing data from the qualitative evaluation questionnaires were not imputed. Dependent samples t-tests were performed to assess whether changes from pre- to post-treatment and from pre-treatment to follow-up were statistically significant. Dependent t-tests comparing post-treatment to follow-up scores were also performed to test maintenance of improvements. The effect sizes of within-group changes between the measurement points were calculated as Cohen’s *d*, including 95% confidence intervals [41]. Means, standard deviations, and standard errors of t-tests and effect sizes were pooled from five imputations using “Rubin’s rules” [42] and the small sample correction for pooled degrees of freedom [43]. To test for differences in insomnia symptom severity level (remission, subthreshold, moderate or severe) at pre-treatment, post-treatment and follow-up assessments, the Wilcoxon signed ranks test was performed, with missing data replaced by last observation carried forward. Treatment evaluation forms included only single item questions and qualitative data, and data from these forms is reported descriptively only.

## Results

As can be seen in Table 2, participants were mainly female, reported on average 3.3 current comorbid psychiatric problems (in addition to ADHD and sleep problems), a majority used stimulant medication(s) for their ADHD, all patients screened positive for insomnia, a majority also screened positive for one or more additional sleep disorders including sleep phase problems, and about two in five used sleep medications.

**Table 2:** Participant demographics, clinical profile and medication use at pre-treatment

Variable	Total *n* = 19
Age	
Mean (Range)	37.0 (19–57)
Sex	
Female	13 (68%)
Marital Status	
Single	9 (47.4%)
Married/registered partnership/in a relationship	8 (42.1%)
Divorced/widow/widower	1 (5.3%)
Other	1 (5.3%)
Educational Level	
Primary school	1 (5.3%)
Secondary school	9 (47.4%)
University	9 (47.4%)
Occupation	
Working/studying/self-employed	9 (47.4%)
On sick leave, disability pension etc	8 (42.1%)
Other	2 (10.5%)
Pre-treatment ADHD severity^a^	
Overall, Mean (SD)	43 (13.6)
Inattention subscale, Mean (SD)	24 (7.2)
Hyperactivity/impulsivity, Mean (SD)	20 (7.9)
ADHD-subtype	
Predominantly hyperactive/impulsive	4 (21%)
Predominantly inattentive	6 (32%)
Combined subtype	7 (42%)
NOS	1 (5%)
Current comorbid psychiatric symptoms	13 (68%)
Comorbid psychiatric problems in the past year^e^	
Depression	15 (79%)
Anxiety	13 (68%)
Panic attacks	8 (42%)
Excessive worry	8 (42%)
Post-traumatic stress	4 (21%)
Specific phobia	4 (21%)
Intrusive thoughts and/or impulses	4 (21%)
Psychosis	1 (5%)
Manic episodes	1 (5%)
Alcohol and/or substance abuse/addiction	1 (5%)
Other	3 (16%)
Number of comorbid psychiatric problems in the past year	
Mean (SD)	3.3 (2.6)
Sleep Disorder Screening^b^	
Insomnia	19 (100%)
Nightmares	15 (79%)
Circadian rhythm sleep disorder	14 (73%)
Sleep apnea	14 (73%)
Restless legs/Periodic limb movements disorder	10 (53%)
Narcolepsy	4 (21%)
Daytime impairment (irritation, concentration difficulties etc.)	18 (95%)
Years with Sleep Problems^f^	
Mean (range)	15.3 (1.5–40)
Pre-treatment Insomnia severity^c^	
Mean ISI score (SD)	15 (3.9)
Clinical insomnia, severe (22–28 points)	1 (5%)
Clinical insomnia, moderate severity (15–21 points)	11 (58%)
Subthreshold insomnia (8–14 points)	6 (32%)
No clinically significant insomnia (0–7 points)	1 (5%)
Circadian type^dg^	
Extreme morning type	1 (5%)
Morning-type	0 (0%)
Intermediate type	2 (11%)
Evening-type	3 (16%)
Extreme evening-type	12 (63%)
Current stimulant use, n (%)	15 (79%)
Stimulants^e^	
Methylphenidate	7 (37%)
Dextroamphetamine	4 (21%)
Lisdexamfetamine	7 (37%)
Current sleep medication use, n (%)	8 (42%)
Sleep medications^e^	
Melatonin	4 (22%)
Z-drugs	2 (11%)
Antipsychotics	3 (16%)
Antihistamines	2 (11%)
Current use of other medication, n (%)	11 (58%)
Other medications^e^	
Anticonvulsant	3 (16%)
Antidepressant	7 (37%)
Other	8 (42%)

^a^Adult ADHD Self-Report Scale [36]

^b^Sleep-50 [32]

^c^Insomnia Severity Index [34]

^d^Morning-eveningness question from the Karolinska Sleep Questionnaire [32]

^e^Each individual may mark more (or less) than one

^f^ *n* = 12

^g^ *n* = 18

### Data attrition

The ISI was completed by 18 (95%) of the 19 participants at post-treatment and 17 (89%) at 3-month follow-up assessments. The corresponding numbers for ASRS was 18 (95%) and 16 (84%). Missing items for these questionnaires were less than 1% (0.4–0.9) at each assessment point.

Treatment evaluation forms were handed in by 13 patients. Missing items were more common in these questionnaires, i.e. 7%.

### Symptom related outcomes

Means, standard deviations, and within group effect sizes, including confidence intervals, for symptom related outcomes at all assessment points are reported in Table 3.

**Table 3:** Means, standard deviations and effect sizes for symptom related outcomes, observed data with imputations, n = 19

Measure	Pre-treatment		Post-treatment		Three-month Follow-up		Within Group Effect Size *d* (95% CI)		
	*M*	SD	*M*	SD	*M*	SD	Pre-post	Pre-Fu3	Post-Fu3
ISI	15.4	4.2	10.9	6.0	8.6	4.7	0.84*** (0.31–1.37)	1.52*** (0.87–2.18)	0.42 (−0.12–0.95)
ASRS (total)	43.2	13.2	40.2	15.7	38.7	13.0	0.20 (−0,12–0.51)	0.34* (0.05–0.62)	0.09 (−0.10–0.29)
Inattention	23.7	7.2	22.2	8.1	21.6	6.7	0.19 (−0.01–0.39)	0.31* (0.05–0.57)	0.08 (− 0.18–0.34)
Hyperactivity	19.9	7.9	17.6	8.4	16.5	6.9	0.28* (0.00–0.55)	0.44*** (0.20–0.69)	0.14 (−0.07–0.35)

*ISI* insomnia severity index, *ASRS* adult ADHD self-report scale

*p < 0.05, **p < 0.01, ***p < 0.001

For insomnia severity, intent-to-treat analyses with dependent t-tests showed statistically significant improvements on the ISI from pre- to post-treatment (t (13.63) = 3.96, *p* = .002), from pre-treatment to follow-up (t (13.47) = 7.01, *p* < .0001), but not from post-treatment to follow-up (t (12.74) = 1.73, *p* = .11). On the ISI, the within-group effect size from pre-treatment to post-treatment was *d* = 0.84, and from pre-treatment to follow-up a larger *d* = 1.52.

Two sensitivity analyses were performed. The first was conducted using non-parametric (the Wilcoxon signed ranks test) as opposed to parametric tests, and this analysis was conducted using non-imputed data (i.e. also testing for possible problems due to imputations). This analysis confirmed and somewhat extended the results from the t-tests, indicating that not only were the median ranks for the post-treatment assessment statistically significantly lower than the median ranks for pre-treatment assessment, but also that the median ranks for the follow-up assessment were statistically significantly lower than the median ranks for the post-treatment assessment (*p*’s .031 > .000), thus indicating continued improvement between post-treatment and follow-up. These results then support the initial findings, using non-parametric analyses and non-imputed data. The second sensitivity analysis was conducted to control for possible social desirability in the reporting of insomnia severity, using patient’s score for their overall impression of the treatment as a proxy for social desirability and entered as covariate in an ANCOVA with ISI score at pre- post and follow-up as the dependent variable. This sensitivity analysis showed a statistically significant effect of time (*p* = .001), but the interaction with patient’s overall impression of the treatment was not statistically significant (*p* = .673), indicating that the reporting of insomnia severity was not governed by patients’ treatment satisfaction.

**Fig. 2:**
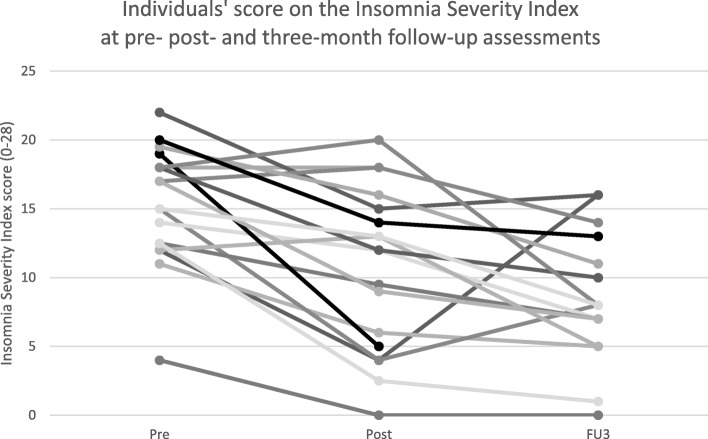
Individuals’ score on the Insomnia Severity Index at pre- post- and three-month follow-up assessments

Figure 2 shows individual ISI scores at pre- post and follow-up assessments. As can be seen, three patients’ insomnia severity did not improve during the treatment period. These three patients had all improved from post-treatment to follow-up assessments. From pre- to follow-up assessments, one patient (5%) had a 4-point increase on the ISI, while at post-treatment assessment this patient had reported lower levels of insomnia.

**Fig. 3:**
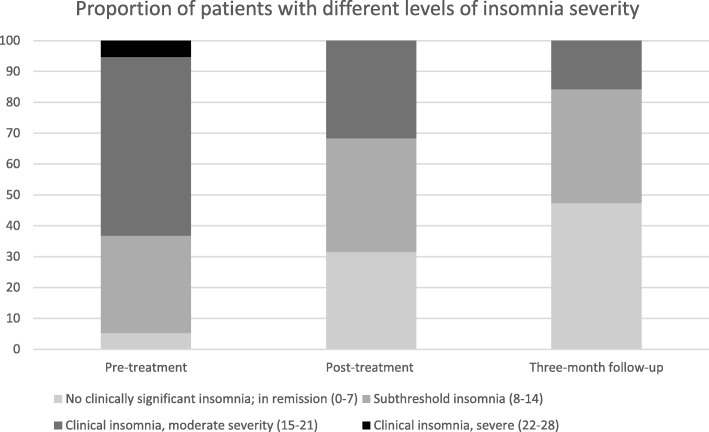
Proportion of patients with different levels of insomnia severity at the three assessment points

Figure 3 shows the proportion of patients at different levels of insomnia severity over the measurement points, with last observation carried forward for the two patients with missing data described above. The results from the Wilcoxon signed ranks tests indicate that patients moved from a higher to a lower severity level, i.e. the median post-treatment ranks were statistically significantly lower than the pre-treatment ranks (*Mdn* Pre = 3, *Mdn* Post = 2, Z = .000, *p* = .003), and that the lower level was maintained at three-month follow-up, i.e. median follow-up ranks were significantly lower than the pre-treatment ranks (*Mdn* Pre = 3, *Mdn* FU = 2, Z = 8.000, *p* = .000), but did not differ from the post-treatment ranks (*Mdn* Post = 2, *Mdn* FU = 2, Z = 15.000, *p* = .166).

For ADHD-symptoms, intent-to-treat analyses with dependent t-tests showed significant improvements on the ASRS from pre-treatment to three-month follow-up (t (8.87) = 2.77, *p* = .02, *d* = 0.34) but not from pre- to post-treatment (t (11.77) = 1.38, *p* = .20) or from post-treatment to follow-up (t (15.45) = 1.00, *p* = .33). The within-group effect size from pre-treatment to follow-up was in the small range.

Post hoc analyses for ASRS subscales (see Table 3) showed statistically significant changes on the hyperactivity subscale between pre-treatment and post-treatment (t (17)=2.15, *p =* .05, *d* = 0.28), and between pre-treatment and three-month follow-up assessments (t (17)=4.14, *p =* .001, *d* = 0.44). At three-month follow-up, patient average on the hyperactivity subscale ended up just below the cut-off for probable ADHD-diagnosis (i.e. < 17). The inattentive subscale did not show a statistically significant change from pre-treatment to post-treatment assessment, but a statistically significant change was seen between pre-treatment and three-month follow-up assessment (t (17)=2.54, *p =* .02, *d* = 0.31).

#### Patient compliance and satisfaction with treatment

- **Proportion of patients dropping out of treatment.** Two patients (11%) dropped out of treatment. One of these patients attended 1 session and the other 2 sessions before not showing up, and subsequently declining further participation, stating personal reasons for their discontinuation.
- **Average number of sessions attended.** Patients attended an average of 7.2 out of 10 sessions (median = 8). Patients who did not drop out of treatment attended an average of 7.9 sessions (median = 8). Most missed sessions were due to illness or more pressing engagements, such as work or doctor’s appointments. Some patients had a hard time making it to the sessions due to difficulties planning and organizing. Patients who missed a session without a cancellation were telephoned by the therapist as soon as possible after the session, in addition to their regular telephone appointment.
- **Patients’ satisfaction with treatment.** All thirteen patients who completed the treatment evaluation form would recommend the intervention to a friend with ADHD and sleep problems. For the overall impression, twelve patients answered and six patients rated 6 (very good), five rated 5 (good), and one rated 4 (quite good).
- **Subjectively reported most important treatment components.** 11 patients reported on most useful treatment components, and the three most commonly reported were: systematic use of light in the morning (reported by eight participants, 73%) and darkness in the evening (eight participants, 73%), and the use of a sleep schedule (seven participants, 64%). Other treatment components that were mentioned were Bedtime routines, the 17 + 7 rule, Relaxation, Stimulus Control, Morning routines, Psychoeducation about sleep, No napping, Engagement in physical or outdoor activity during the day, Worry time, Limit the use of tablets/telephones in bed and other Sleep hygiene practices.

## Discussion

In this pragmatic within-group pilot study, a novel group intervention based on CBT for insomnia, adjusted for patients with ADHD (CBT-i/ADHD), was investigated in a clinical setting. We conducted a preliminary evaluation of possible effects of the intervention, and gauged patient compliance and satisfaction, at a specialist psychiatric out-patient clinic. We found that insomnia severity improved significantly with a medium to large within-group effect from pre-treatment to post-treatment assessments, and was stable at three-month follow-up. We also found an improvement in ADHD symptoms at three-month follow-up compared to pre-treatment. In addition, patient compliance with the intervention in a clinical setting was good, since most participants came to an adequate number of treatment sessions. Finally, patients were satisfied with the treatment. Overall, these results point to a promising treatment that is acceptable and feasible in a clinical setting.

The sleep related adjustments made to this treatment manual were made based on the literature on sleep problems in ADHD, and included components targeting insomnia and other sleep and circadian problems such as sleep phase shifts and problems with sleep hygiene and waking up in the morning. Quite recently, Harvey and Buysse suggested a new way of working with sleep problems in youth and potentially other patient groups, the so called TranS-C-approach [44, 45]. Interestingly, the TranS-C approach includes many of the same strategies that were adopted in this treatment, including systematic work with light and other zeitgebers, and wake-up routines. To our knowledge, no evaluation of the TranS-C-approach has been published as of yet, but the Oxford Ward sLeep Solution (OWLS) for psychiatric in-patients [46] is building on similar ideas. A pilot trial of the OWLS was recently published, suggesting the OWLS would lead to improvement in insomnia severity, and fewer days in admission [47]. The OWLS is used in patients with different psychiatric problems, pointing to the possibility that other patient groups might also benefit from some of the adaptations used in the CBT-i/ADHD. For instance, dysregulation of the circadian system has been implicated in both patients with bipolar disorder [48] and psychotic disorders [49] so these patient groups might also benefit from strategies aimed at stabilizing the circadian rhythm. However, although our treatment contains components aiming to shift and stabilize participants’ circadian rhythm, we do not measure circadian rhythmicity, and thus do not know whether it changes following this treatment. Future studies are needed to investigate this aspect.

Other alterations were made to increase the likelihood for patients with ADHD to achieve the sleep related behavior changes that are central to this treatment (e.g. keeping constant bedtimes, timing light exposure, and doing relaxation exercises). To this end, we added strategies for organizing and planning in the first session, drawing from the Safren manual on CBT for ADHD [37]. We also added telephone calls from a therapist between each treatment session. Neither of these components is commonly included in a CBT-i-treatment. In addition, we increased the number of session and shortened the length of sessions. Which – if any – of these adaptations are important, we do not know. The present study was not designed to test the usefulness or relative importance of different treatment components, but that is an important area for future research studies.

The effects on insomnia severity in the present study are in the medium to large range (post-treatment, *d* = 0.84; three-month follow-up, *d* = 1.52), and the proportion of patients in remission increase over time (32% at post-treatment, and 42% at three-month follow-up) while the proportion of patients with moderate or severe insomnia decreased over time (63% at pre-treatment, 32% at post-treatment, and 16% at three-month follow-up). Considering patients have at least one other disabling psychiatric disorder (i.e. ADHD) and most have additional comorbidities, it is encouraging that patients, after this behavioral intervention, reported improvements in insomnia severity and proportion of remitters after treatment comparable to previous studies with non-ADHD populations [29, 38].

In addition to the improvement seen in insomnia severity, ADHD-symptoms improved slightly between pre-treatment and three-month follow-up assessments, which is especially interesting considering about 80% of patients already have pharmacological treatment for their ADHD. The improvement seen in this pilot is small in effect (*d* = 0.34), and due to the within-group study design it cannot be causally attributed to the treatment, but to our knowledge this is the first study showing possible improvements on ADHD symptoms after a behavioral sleep intervention. Post-hoc analyses indicated that the change was larger on the hyperactivity sub-scale (*d* = 0.44), and at three-month follow-up patient average actually fell below the cut-off for probable ADHD on this sub-scale (i.e. 17 points). This is especially interesting since arousal is an important aspect of sleep, and included in several of the theoretical models of insomnia (e.g. the cognitive behavioral model of insomnia [50] and hyperarousal theory [51]). Inability to control arousal or wrongly timed arousal can cause acute insomnia, and can be a contributing factor for chronic sleep problems. In ADHD, hyperactivity is a core symptom which has been associated with insomnia [52]. The decrease in hyperactivity symptoms seen in the patients in this study could conceivably be indicative of an amelioration of negative consequences of poor sleep with symptoms similar to ADHD-symptoms, and/or a decrease in arousal, possibly associated with a shift in the circadian rhythm, following the treatment. These possible mechanisms are intriguing and warrant further study. Although the decrease in ADHD-symptoms is smaller than that produced by psychological treatments targeting ADHD-symptoms (e.g. [4, 5]) it is nonetheless very encouraging.

Whether these results would hold in a randomized trial remains an empirical question. Since this is only a small pilot with no control group, results should be interpreted with caution. Apart from this basic methodological weakness, a couple of limitations should be noted; sleep problems are not diagnosed but only screened for, there is no objective measure of sleep and no sleep diary data; pharmacological interventions during the study period are not controlled for, neither for insomnia nor for ADHD, and we can thus not exclude the possibility that improvements are due to other factors, for instance changes in medication. Indeed, both stabilizing the use of sleep medications, and adjusting ADHD medication timing, was encouraged in this intervention. However, neither was reported by the participants as the most useful treatment component, indicating that other strategies were perceived by patients as having greater impact on their sleep.

Important strengths of this study are the low intervention drop-out and study attrition. Also, therapists, who were psychologist students with limited clinical experience, could successfully deliver this treatment, suggesting the treatment manual would be useful also to clinicians who are not experts in behavioral sleep medicine. Finally, the fact that the intervention was given in a naturalistic setting to patients at a specialist psychiatric out-patient clinic, makes direct generalization to this patient group possible.

To confirm and elaborate on these positive preliminary results, a randomized trial is currently being conducted at the Department of ADHD, Northern Stockholm Psychiatry (Stockholm, Sweden).

## Conclusions

The CBT-i/ADHD is a newly developed behavioral treatment for patients with ADHD, insomnia and other sleep problems, based on CBT-i and with adjustments to account for problems specific to the ADHD-population. Insomnia severity was reduced with medium to large within-group effect size from pre-treatment assessment to post-treatment and follow-up assessments. A small improvement was also seen in ADHD-symptoms at three-month follow-up, and the treatment was feasible in clinical practice. However, since no control group was used, results should be interpreted with caution, and further studies will be needed to create an evidence base for the use of non-pharmacological alternatives to treat sleep problems, with the potential to greatly improve care and quality of life, in patients with ADHD.

## Data Availability

The datasets used and/or analyzed during the current study are available from the corresponding author on reasonable request.

## Abbreviations

ADHD: Attention deficit hyperactivity disorder
ASRS: Adult ADHD self-report scale
CBT: Cognitive behavioral therapy
CBT-i: Cognitive behavioral therapy for insomnia
ISI: Insomnia severity index
